# Influence of Imagery Training on Adjusting the Pressure of Fin Swimmers, Improving Sports Performance and Stabilizing Psychological Quality

**DOI:** 10.3390/ijerph182211767

**Published:** 2021-11-09

**Authors:** Hsiao-Hsien Lin, Tzu-Yun Lin, Ying Ling, Chih-Cheng Lo

**Affiliations:** 1 School of Physical Education, Jiaying University, Meizhou 514015, China; 2 Department of Sport Information and Communication, National Taiwan University of Sport, Taichung City 404401, Taiwan; lincoach8@gmail.com; 3 Institute of Physical Education and Health, Yulin Normal University, Yulin 537000, China; g169168@yahoo.com.tw; 4 Department of Industrial Education and Technology, National Changhua University of Education, No. 2, Shi-Da Road, Changhua City 500, Taiwan

**Keywords:** fin swimming, imagery training, imagery framework, avoidance mentality

## Abstract

This study analyzed the effects of imagery training on athletes’ imagery ability, physical anxiety and athletic performance. This study employed a mixed research approach. Snowball sampling was used to select 55 fin swimmers with imagery training experience and formal competition participation. Basic statistics were obtained, and Pearson product-moment correlation coefficient (PPMCC) analysis was performed using IBM SPSS Statistics for Windows, Version 26.0, and the results were compared with the opinions of three experts and were tested using multivariate validation methods. The results revealed that although imagery training can help athletes improve their performance and significantly reduce their anxiety during the competition, athletes can still make mistakes due to internal and environmental factors and even have negative thoughts that lead to their reduced likelihood of competition participation. By strengthening strategic and technical imagery training, we can help our fin swimmers perform at a higher level, achieve their goals, and improve overall satisfaction with their competition process and performance.

## 1. Introduction

Fin swimming is similar to the way cetaceans swim [1]. It uses the bionic fins worn on the swimmer’s feet to drive the waist, hips, and thighs to propel the trunk forward [2]. Although this movement pattern looks elegant, it is fast and requires considerable skill [3]. After years of promotion by more than 130 organizations in more than 100 countries worldwide [1,4], it has become one of the most appreciated global sports [1,4,5].

Although the sport of fin swimming has been developed for more than 60 years, it is still in the nascent stage compared with traditional sports such as track and field. Although the development of the sport in Asia has been slower than that in the European region (with a developmental period of almost 20 years) [4], the sport has become more successful with the improvement of education, training, technology, and equipment [5]. However, despite the improvement of training skills, the novelty and uniqueness of fin swimming postures, and the effective learning and imitation of fin swimming in Asia [2], many obstacles are required to be overcome to enhance athletes’ performance, improve their behavior, and increase their efficiency through relying only on their individual behavior and imitation ability. This sport requires the use of fins; the heavy fin on the ankles needs to be driven forward by the force of the body trunk through marked and repetitive flexion and extension movements. If the athlete does not perform these movements in the correct manner or without professional guidance and assistance in training, the lower back muscles and sciatic nerve may be injured [3,6]. Furthermore, fin swimmers rely on a breathing tube or air cylinder while moving forward on the surface or in the water. The flexibility of the rib cage [7] and the strength of respiratory muscles [8,9] are crucial to the performance of the sport [3,10]. Although the performance and process of fin swimming are elegant and pleasing to the eye, this sport is highly effort-intensive [11]. Athletes need to maintain a high level of concentration during training, which can stabilize the athletes’ performance, improve their training effectiveness, and prevent injuries. However, training in a high-stress environment all the time can lead to increased anxiety and physical and mental effects [12,13]. Therefore, identifying movement patterns that reduce anxiety and stabilize performance [12,13,14] can help athletes improve physical fluency and performance during training or in competitions [14,15], ultimately leading to positive results.

Having adequate knowledge and skills in sports can improve athletic performance [16,17] and effectively improve physical and mental health [18,19]. Adequate athletic knowledge and skills can enhance performance [17] and effectively improve physical and mental health [18,19]. However, the competitive environment of fin swimming is fast-paced and high-tension, and the athletes’ movement and maneuvers are limited by the regulations of equipment and behavior patterns of the sport [1,4,5]. During swimming, the body moves in a particular way [7] and at a fast pace, requiring a strong and durable physical quality to avoid injuries [3,6,10]. In addition, athletes need to maintain a steady and high level of concentration during athletic training or competition to achieve better performance. As a result, fin swimmers are always in a state of high tension and intensity, which puts a lot of psychological stress on them [20]. Without strong resilience and willpower, athletes will not be able to overcome their fears [21], which may lead to poor performance in training or competition, or even physical and mental trauma.

Imagery training is also an adaptive mental skill that can be used to help subjects improve behavioral imagery, self-management of stress, and attention problems [19]. It aids and enhances the memory of physical movements and behaviors by replaying the memory in the brain to simulate and reconstruct the scene of the competition. Imagery training simulates actual behaviors and feelings, accumulates real experiences, and stores them in the consciousness [20], which helps to improve mental abilities, enhance motor fluency and autonomy [21,22], and improve performance failure and psychological stress in highly stressful and intense environments for athletes. In addition, imagery training can help subjects to re-establish motivation for participation [23]. Positive motivation to participate helps to establish an appropriate level of arousal to control emotions, reduce anxiety and tension, and stabilize the mental ability for sport, which in turn improves athletic performance [24]. Studies have demonstrated that imagery training for 3–7 weeks [25,26], at least once a day or twice a week [27,28], has the ability to improve athletic cognition and motivation [29], reduce negative emotions in competition [24,30], stabilize and improve athletic performance [31,32,33], and have a predictive effect on final athletic performance.

To summarize, although the sport of fin swimming is highly appealing to the eye and the introductory movements are easy to imitate [2], to improve athletic performance, reduce injuries during competitions, and achieve optimal athletic performance [3,6], the strengthening of psychological capacity and the reduction of anxiety are necessary [15,16]. Imagery training may improve athletic performance and help fin swimming athletes achieve positive results. Although many studies have applied imagery training to other sports and investigated its effectiveness for improvement in sports medicine [19,29], sports performance [20,30], and psychological aspects [26,30,31,32,33], most studies in the field of fin swimming have only investigated sports physiology [11], psychology [5], exercise dynamics [3,7,11], and sports behavior [2]. Therefore, to address this research gap, the present study analyzed the effects of imagery training on the imagery ability, physical anxiety, and sports performance of individual fin swimmers during the competition and its effectiveness for optimizing fin swimming performance and achieving the goal of the internationalization of fin swimming.

In summary, although there is evidence in the literature that imagery training has a significant effect on improving athletes’ skills and fluency, enhancing motivation and cognition, and alleviating physical and mental stress, no studies have been conducted on fin swimming. Therefore, referring to the theory of imagery training to determine the experimental subjects and procedures, this study aimed to investigate whether imagery training had an effect on imagery ability, physical anxiety, motor performance, and physical and mental health in fin swimming.

## 2. Literature Review

### 2.1. Imagery Training and Imagery Ability

Imagery simulates actual experience, an experience accumulated through the regeneration of mentally created sensations [34,35,36]. The imagery intervention aims to achieve the desired goal of the mind through imagery ability [15]. Motor imagery refers to the production of images based on motor perception, in which the visual, sensory, auditory, and kinesthetic cues of the subject matter are synthesized into images in the mind through a planned process based on the individual’s memory of real sensations and experiences [37,38], and these cues are rehearsed in a “deliberate” manner in the mind [39].

Imagery ability refers to the quality of an individual’s imagery [37], which contains two major keys: clarity and control. The clarity and control of the imagery content affect the quality of the imagery [37,40], and the closer the imagery content is to the actual situation, the higher is the imagery quality and ability [26,41]. Sports performance is enhanced through imagery training, and the design of the program, and performance can be judged based on the current performance, expected goal, comparison with the previous level, and error rate [42].

Imagery simulation can enhance exercise effectiveness by enabling one to imagine the upcoming movement pattern or technique [43,44], and the measures of imagery training can predict individuals’ imagery outcomes [45], effectively enhancing the neural feedback required for exercise, reducing errors, and improving stability and performance [23,24,46]. The higher is the level of engagement in imagery training, the higher is its effect [26,41,47,48]. In summary, imagery training can improve the imagery of athletes, and this method should be applied to improve the competition imagery of fin swimmers. The effect of imagery training on the imagery of fin swimmers should be investigated to determine whether imagery training exerts the same effect on the mental status of fin swimmers during the competition.

### 2.2. Imagery Training and Physical Anxiety

Physical anxiety is an emotion formed by the presence of various negative feelings such as stress, anxiety, uneasiness, worry, and fear [49,50], and emotional stress is a key factor contributing to success or failure in a sports competition [51]. Anxiety is generated by the pressure of the competition environment [52], and it is the result of stress generated by the subjective understanding of an individual and activates autonomic emotion responses [53]. The state anxiety is a negative emotion, such as nervousness, anxiety, worry, anxiety, and uneasiness, that occurs in a particular situation [54].

Anxiety interferes with athletic performance [55], and its negative effects include physiological symptoms, such as muscle tension, rapid heartbeat, stomach tension and sweaty palms, which can affect performance and outcome during the competition [56]. However, anxiety can exert positive effects, and low levels of anxiety can lead to an increase in self-competency and a change in the status quo in an effort to catch up with a self-imposed goal [57]. Imagery training can be effective in mental skills training [58], reducing race anxiety, and improving self-confidence [59]. Imagery training through mental training programs, goal setting, and integrating relaxation techniques and self-talk skills [30] is effective in alleviating anxiety.

Studies have confirmed the effectiveness of mental imagery in reducing anxiety behaviors [60] and pain in athletes, ensuring that athletes are mentally and psychologically prepared [61]. Different levels of exposure have different effects on reducing anxiety [62]. The higher is the level of exposure, the higher is the effect [61,62].

In summary, imagery training can improve physical anxiety during competition, and this approach should be applied to improve physical anxiety in fin swimmers during the competition. Therefore, the effect of imagery training on the anxiety of fin swimmers should be investigated to determine whether imagery training can reduce the anxiety of fin swimmers.

### 2.3. Imagery Training and Motor Performance

In the learning of individual skills, imagery can generate basic cognitive processes for motor learning and performance enhancement [55]. Imagery training for athletes can stimulate brain areas involved in actual movements through the conscious use of imagery, resulting in neurological and behavioral similarities with actual experience [56] and helping athletes to improve their psychological quality and athletic performance [26].

In sports competitions, technical, physical, and psychological qualities are the major influences on winning and losing [57], and the manifestation of an athlete’s mental skills on the spot is a key factor determining victory [42].

Imagery training significantly improves skill acquisition and performance as well as increases muscular stability [41] and enhances skills learning [32]. Pre-competition tactics, in-sport corrections, improved decision-making, emotional regulation, imagining victories, alternative strategies, excitement, basic movements, future improvement plans, encouragement, persistence, and execution rate of movements performed at the beginning [46,58] illustrate the current state of athletic performance fluency. Imagery training can improve athletic performance during the competition, and this method should be applied to improve the competitive performance of fin swimmers. Therefore, examining the effect of imagery training on physical anxiety in fin swimmers will reveal whether imagery training can improve the performance of fin swimmers during competition.

### 2.4. Effect of Imagery Ability and Physical Anxiety on Athletic Performance

The performance of athletes may differ between training and competition periods [59]. Due to the pressure of the competitive environment [45], athletes experience stress and anxiety [46], which lead to nervousness, anxiety, worry, and anxiety [45] and subsequent muscle tension, sweating, and stomach discomfort [51], resulting in low athletic performance [50].

The effectiveness of sports performance is related to proficiency in athletic skills [63], which is affected by the imagery ability of athletes [59,60,61,62,63]. Highly effective imagery ability can lead to greater clarity in the individual’s process of learning experience [37,38], and high-quality imagery content can lead to imprinting the entire learning experience in the mind [39], with higher imagery quality [26,41] resulting in higher sports performance [42,59,60,61,62,63].

The physical and mental condition of athletes affect performance during the competition, and the effectiveness of imagery ability can influence performance proficiency, which, in turn, affects performance effectiveness. Therefore, this theory can be used to study the correlation among the imagery ability, physical anxiety, and athletic performance of fin swimmers.

## 3. Methodology

### 3.1. Research Process and Assumptions

**Figure 1:**
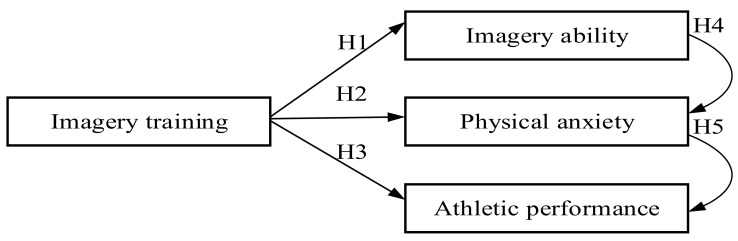
Research framework.

Enhancing athletic performance and achieving excellence are the ideals and goals of athletes in sports [21]. However, the intense competition [59] and the high intensity of sports can affect the psychological status of athletes in the sports arena, resulting in stress, anxiety, and loss of concentration [45]. Physiologically, problems of body tension, sweating, and stomach discomfort may occur [51], which can affect athletic performance [50]. Imagery training can improve athletic performance by deepening athletic memory [37,38,39], enhancing imagery ability, strengthening psychological quality, and stabilizing athletic performance during the competition [42,59,60,61,62,63]. Therefore, this study analyzed the effects of imagery training on the imagery ability, physical anxiety, and sports performance of fin swimmers based on the aforementioned theoretical model to identify ways to stabilize and improve fin swimmers’ competitive sports performance. The framework of the study is shown in Figure 1.

According to the aforementioned literature, five research hypotheses are proposed as follows:

**Hypotheses** **1** **(H1).** *Imagery ability of fin swimmers changes after imagery training.*

**Hypotheses** **2** **(H2).** *Physical anxiety of fin swimmers improves after imagery training.*

**Hypotheses** **3** **(H3).** *The fin swimmer’s athletic performance improves after the imagery training.*

**Hypotheses** **4** **(H4).** *A significant correlation is present between imagery ability and athletic performance.*

**Hypotheses** **5** **(H5).** *A significant correlation is present between physical anxiety and athletic performance.*

### 3.2. Study Population and Research Setting

We developed and edited a questionnaire based on the literature related to fin swimming and imagery training [1,2,3,4,5,6,7,8,9,10,11,12,13,14,15,16,17,18,19,20,21,22,23,24,25,26,27,28,29,30,31,32,33,34,35,36,37,38,39,40,41,42,43,44,45,46,47,48,49,50,51,52,53,54,55,56,57,58,59,60,61,62]. We used Taiwan as a case study to understand the effects of imagery training on the imagery ability, physical anxiety, and athletic performance of fin swimmers. Although fin swimming has been promoted in Taiwan for many years, the number of participants has been low due to the limitations of expensive equipment, exercise style, physical condition, and duration of exercise career, and an adequate sample size could not be obtained due to the impact of the COVID-19 pandemic. Therefore, the investigators selected 55 volunteers for the survey based on the study design in the relevant literature [14,61,62]. Because scientific research needs to be conducted with adequate theoretical support, rarity or novel research areas are relatively weak in terms of the theoretical foundation. Adopting a complex research approach, complementing the breadth of research with quantitative research methods [64,65], and increasing the depth of research with qualitative research [66] can compensate for research methodological or theoretical shortcomings [67].

The questionnaire was compiled by referencing the relevant literature [14,15,16,17,18,19,20,21,22,23,24,25,26,27,28,29,30,31,32,33,34,35,36,37,38,39,40,41,42,43,44,45,46,47,48,49,50,51,52,53,54,55,56,57,58], and the content of the questionnaire was then verified by three coaches with national sports team coaching experience and academics in the fields of exercise physiology and psychology, as shown in Table 1.

Three questionnaires, revised after validation analysis, were then distributed, and the reliability of the questionnaires was examined using the SPSS version 26.0 statistical software (company, city, state abbreviation if USA, country). The final questionnaire had a high reliability of Cronbach’s α of 0.7 or higher [42].

### 3.3. Procedure, Analysis, and Limitations

The literature suggests that improvement is facilitated by using imagery training for more than 2 weeks, at least twice per week. With the adequate and reasonable design of pre-test, intervention, and post-test processes [68,69,70], a rigorous research structure, and diversified research methods [64,65,66,67,71,72,73], sufficient analytical evidence can be obtained even if the final data is based on 9–12 respondents [74,75,76]. An even more accurate answer can be obtained if we can further apply qualitative research methods, collect the opinions of experts, scholars, and participants, categorize and summarize the data in a stringent manner, and compare the contents using multivariate verification analysis [77,78,79,80,81].

This study was restricted by environmental safety considerations of the COVID-19 pandemic, limited human and financial resources, and the small number of participants currently participating in fin swimming. To obtain a rigorous and representative sample for analysis, subjects were required to have participated in an official fin swimming competition at the regional level or above and to have won an award.

Respondents were selected through purposive sampling. According to the rules and regulations of monofin swimming and competition instructions, swimmers must be at least 13 years old. Therefore, the target population was defined as athletes who were at least 13 years old, had at least one year of experience in imagery training, and had at least ranked among the top 8 finishers in a national competition. All subjects participated in the study only after they had received adequate instructions and confirmed their willingness to participate in the experiment. The video content used in the training was developed by the coaches and the imagery training experiment was conducted at least twice a week for 6 months without affecting the coaches’ and players’ training program goals. The experimental process was assisted by the coaches of each team and was followed by post-experimental surveys after the stages of imagery intervention, mock competition test, imagery intervention, pre-competition preparation, and national competition. Due to the risk of epidemic transmission, the pre-test, intervention, training, and post-experimental phases were conducted through a remote communication and online questionnaire platform. The initial questionnaire survey was conducted by the intentional sampling method for the collection of subjects. At later stages, taking into account factors such as continuity of training and injuries, we adopted the snowball sampling principle and asked participants to recommend potential respondents to increase the sample size. The final sample included 55 athletes (27 male and 28 female participants) who were aged at least 13 years, and the oldest respondent was 28 years; they had participated in fin swimming for at least 1 year and up to 8 years, with a mean of 3.8 years and an SD of 0.994.

This study adopted a mixed research approach. Basic statistics were used to examine the cognitive level of imagery ability, body anxiety, and athletic performance during the competition after fin swimmers had received imagery training, and the questionnaire was analyzed using PPMCC to determine the effect of imagery ability and body anxiety on athletic performance.

**Figure 2:**
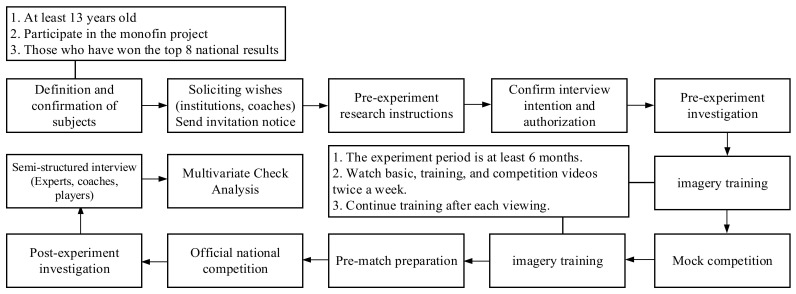
Experimental design specifications and flowchart.

The experts were then interviewed using the VooV Meeting (Android/iOS) videoconferencing program to obtain their personal opinions of the questionnaire. Then, all information was categorized and integrated after compilation, organization, and analysis to construct the report in a robust manner [68,69,70], and the data were examined through multivariate validation analysis. An illustration of the experimental design and process of the study is shown in Figure 2.

### 3.4. Measurements

According to the literature, imagery training can reduce athletes’ error rate, improve athletic performance and physical stability [15,16,17,18,19,20,21,22,23,24,25,26,27,28,29,30,31,32,33,34,35,36,37,38,39,40,41,46,55,56,57,58], reduce physical and psychological barriers [29,42,43,44,45,46,47,48,49,50,51,60,61], increase stress resistance and willpower [46,48], and ultimately improve athletic performance [26,32,41,46,55,56,57,58]. Therefore, the researchers hypothesized that imagery training should have the same effect on the imagery ability, physical anxiety, and athletic performance of fin swimmers. Information and practical questions from experts and coaches were also gathered, and the characteristics of the sport of fin swimming, such as its operation, spectator value, and competition pace, were taken into account. Therefore, we compiled questionnaires on imagery ability [15,16,17,18,19,20,21,22,23,24,25,26,27,28,29,30,31,32,33,34,35,36,37,38,39,40,41,46,55,56,57,58], physical anxiety [29,42,43,44,45,46,47,48,49,50,51,60,61], and athletic performance [26,32,41,46,55,56,57,58] based on the abovementioned literature and the information provided by experts and coaches. After that, experts and coaches were invited to review the validity of the content and confirm the suitability of the topic and the target audience before the analysis was conducted.

The questionnaire adopted a 5-point Likert scale, with a score of 1 being very dissatisfied and 5 being very satisfied. After the content was edited with reference to the literature, three experts examined the content, and SPSS 26.0 statistical software was then used for statistical analysis. The Kaiser–Meyer–Olkin (KMO) value of >0.06 and a *p* value of less than 0.01 in Bartlett’s test indicate that the scale is suitable for continuous factor analysis [81]. An α value greater than 0.60 indicated that the questionnaire had high reliability [82]. as shown in Table 2.

Scholars have pointed out that the number of subjects for the pretest should be at least three times the number of items in the scale with the greatest number of questions in the questionnaire [83], and no less than 100 [84]. Due to the limitation that the study population had to be the top eight athletes in the country, 100 questionnaires were collected for the pretest. The questionnaire options were different genders; less than 1 year, 1–3 years, 4–5 years, and more than 5 years of swimming experience in fin swimming; frequency of imagery training such as infrequent to regular. 

Nineteen questions were compiled after referring to the literature on imagery ability [15,16,17,18,19,20,21,22,23,24,25,26,27,28,29,30,31,32,33,34,35,36,37,38,39,40,41,46,55,56,57,58]. The overall KMO of the 19 questions was 0.0.809, Bartlett’s approximate χ2 value was 6944.341, and the degree of freedom (df) was 171, with significance of 0.000 (*p* < 0.001), indicating that the questions were suitable for factor analysis. The analysis revealed that the explained variance of the scale was 19.3%, 18.8%, 15.9%, 13.9%, and 7%, with a total explained variance of 74.9%. All the questions were retained after factor analysis and considering the understanding of the actual state of economic development. The scale dimensions were strategy (four questions), proficiency (four questions), skill (four questions), emotion (four questions), and goal (three questions), with 0.95, 0.95, 0.95, 0.95, 0.95, and 0.95, respectively. Therefore, the scale had sufficient representativeness.

After referring to the literature on body anxiety [29,42,43,44,45,46,47,48,49,50,51,60,61], a total of six questions were compiled. The questionnaire had KMO > 0.700, a Bartlett’s approximate χ^2^ value of 108.231, df of 15, and significance of 0.000 (*p* < 0.001), making it suitable for factor analysis. The explained variance of the scale was 45.3% and 10.1%, and the total explained variance was 55.4%. All of these items were retained after factor analysis and considering the understanding of the actual state of economic development. The scale had two dimensions: psychological anxiety (four questions) and physical anxiety (two questions), with 0.70 and 0.70, respectively. Therefore, the scale had sufficient representativeness.

A total of eight questions were compiled after referring to the literature on athletic performance [26,32,41,46,55,56,57,58]. The questionnaire had KMO > 0.758, a Bartlett’s approximate χ^2^ value of 69.562, df of 6, and significance of 0.000 (*p* < 0.001), making it suitable for factor analysis. The explained variance of the scale was 57.2%, and the total explained variance was 57.2%. Only motor performance was named and was 0.71. Therefore, the scale had sufficient representativeness.

### 3.5. Background

The majority of the participants occasionally used (33%) imagery training techniques to supplement their fin swimming training, the majority of the participants were female (51.5%), and the majority had 4–5 years (31%) of fin swimming training and competition experience, as shown in Table 3.

### 3.6. Ethical Considerations

The main objective of this study was to investigate whether imagery training could affect the imagery ability, body anxiety, athletic performance, and physical and mental health of fin swimmers. Although the researchers know, human and animal experiments require an ethical review process. However, the subject matter and the design of the experimental tests in this study did not use invasive treatments or other medical experimental practices to comply with the requirements of human and animal experimentation for ethical review procedures.

There were five steps in the sampling phase of the study, during which three interviews were conducted: (1) After soliciting the wishes of the participating organizations, coaches, and respondents, a pre-test questionnaire was administered to collect information about the athletes’ status and feelings at the time. (2) The research theme and process were explained, and the participants were communicated with the purpose of the study to obtain more in-depth information. (3) Without interrupting the coaches’ training plans and needs, instructional videos of different skill levels were provided by the coaches for viewing and analysis, and simulated tests were conducted at the same time. (4) After all the subjects participated in the national tournament of the year, and before the post-competition questionnaires were administered, consent and authorization to be interviewed were obtained again. (5) Based on the analysis of the questionnaire data, the opinions of experts and coaches were obtained, and comparisons and analyses were conducted. Therefore, all respondents understood the study topic, agreed to participate in the study, and authorized the use of the study data without disclosing their personal information.

As such, the data and information used in this study have been authorized by the subjects and comply with the conditions and wishes of anonymity and non-disclosure. Therefore, the study process and design were in accordance with research ethics.

## 4. Results

A total of 55 fin swimmers with top eight national ranking and imagery training were surveyed. The basic background, imagery ability, physical anxiety, and sports performance perceptions of the subjects were analyzed through statistical tests. 

### 4.1. Perception Analysis of the Effect of Imagery Training on the Imagery Ability of Fin Swimmers

The literature states that imagery training can modulate athletes’ competition imagery ability [37,38,39,40,41,42]; thus, it is assumed that imagery training results in changes in the imagery ability of the fin swimmers. The analysis revealed that the imagery ability of the fin swimmers after imagery training contributed to the improvement of precompetition tactics (+0.61), alternatives (+0.61), timely correction of deficiencies (+0.15), imagining a good mood (+0.06), and setting corrective goals (+0.03) during the competition, but the athletic proficiency (−0.35), giving all-out effort despite a bad competition (−0.24), and excelling at an event (−0.23), feeling excited about the event (−0.22), competition details (−0.12), winning praise (−0.15), imagining oneself as the most resilient (−0.15), maintaining confidence (−0.10), being encouraged by mentors (−0.10), overcoming the competition with positive emotions (−0.16), winning medals (−0.13), and maintaining a positive attitude after setbacks (−0.05) showed no changes, as shown in Table 4. The improvement in prematch tactical planning and alternatives during competition was the most obvious, whereas the deterioration in athletic proficiency was the most severe.

Clearly, although imagery training can help athletes more favorably plan their tactics and prepare for a competition, it can also obscure an athlete’s confidence in individual athletic skills during the competition.

### 4.2. Perception Analysis of the Effect of Imagery Training on the Physical Anxiety of Fin Swimmers

The literature suggests that physical anxiety can be improved through imagery training [28,53,54,55,56,57,58,59,60,61,62,63,64]. Therefore, it was hypothesized that the physical anxiety of the fin swimmers would improve after imagery training. The analysis showed that the positive emotion of feeling relaxed (+0.61) was significantly increased, and the negative emotions of feeling anxious (−0.38), nervous (−0.32), tense (−0.54), stomach tension (−0.66), and rapid heartbeat (−0.31) were significantly reduced after imagery training, as shown in Table 5. Among them, the most obvious change occurred in terms of body relaxation, and the greatest effect was resolving negative emotions such as stress and anxiety. 

After imagery training, the negative physical and psychological feelings of the athletes during the competition greatly improved.

### 4.3. Perception Analysis of the Effects of Imagery Training on the Athletic Performance of Fin Swimmers

The literature suggests that the effectiveness of athletic performance can be improved by imagery training [26,30,42,55,56,57,58]. It was hypothesized that the athletic performance of the fin swimmers would improve after imagery training. However, after imagery training, the performance of the fin swimmers improved significantly in terms of satisfactory performance (+0.21), achieving the desired goal (+0.31), and exceeding the previous level (+0.56) and decreased in terms of no mistakes at all (+0.17), as shown in Table 6. Among the questions, performance beyond previous levels scored the highest, whereas zero mistakes in sports performance scored the lowest. 

After imagery training, although the athletes could perform above their normal level, they still made some mistakes in the competition and could not achieve a flawless performance.

### 4.4. Analysis of the Correlation between Imaginative Ability and Physical Anxiety on Sports Performance

The literature suggests that athletes’ performance during training and competition may differ [59], and that the pressure of the competition produces negative physiological and psychological states [45,51] that interfere with athletic performance during competition [46]. Thus, it was hypothesized that imagery ability, physical anxiety, and athletic performance show a significant correlation. However, the analysis revealed that physical anxiety and athletic performance were not significantly correlated (*p* > 0.05).

According to Table 7, overall imagery ability (0.347) showed a significant but low correlation with motor performance (*p* < 0.05), which was consistent with H6. However, a significant low correlation (0.391) was observed between overall imagery ability and exceeding previous levels. Furthermore, strengthening strategic imagery showed a significant moderate correlation (*p* < 0.001) with satisfaction with performance (0.456), achieving the predetermined goal (0.443), and exceeding previous levels (0.368), and the enhancing technical imagery showed a significant low correlation (*p* < 0.05) with satisfaction with performance (0.389) and exceeding previous levels (0.383).

## 5. Discussion

### 5.1. Effect of Imagery Training on the Imagery Ability of Fin Swimmers

The results showed that although imagery training helped the athletes develop effective precompetition tactics and alternatives in advance and correct deficiencies in time to enable them to face problems with a good mindset and set corrective goals, it did not help improve proficiency and confidence. The evidence is not consistent with the study Hypothesis 1.

Because the athletes may still lose due to errors that may occur during the competition, this may lead to a fear of not receiving encouragement and honor. The results of this study are thus inconsistent with those in the literature [43,44,45,46].

The researchers believe that the competitive sport itself, the high stress and intensity of the sport environment, and the need to wear the fin and other apparatuses in the sport of fin swimming add to the physiological and health burden and relying on traditional training models alone may not improve the athletes’ innate physiological deficiencies and enhance their athletic performance. Imagery simulation training is a favorable way that enables athletes to understand, in advance, the difficulties they may face in future competitions and to understand how to respond to them through the use of images or videos to immerse the participants in the situation and through systematically designing, organizing, and planning a simulated learning program for athletes, with the assistance of and explanation from coaches.

However, imagery training is also an intangible framework. Although it allows athletes to find solutions to difficulties faced during the competition, it also ingrains athletes with standardized movements and responses. It is like knowing the end of a race; when an athlete finds a flaw in their athletic performance during a race and knows the final outcome of the race in advance, they may have negative thoughts and abandon unfinished movements or races.

Imagery training can help construct pre-competition tactics and alternatives for fin swimmers, and it allows them to visualize good moods during the competition, correct deficiencies in a timely manner, and identify goals for future improvement or correction. However, in the high-stress sports environment, the enormous pressure can affect one’s ability to focus, resulting in changes in athletic proficiency and subsequently the inability to motivate oneself with positive, optimistic attitudes, and emotions and to give one’s all to the competition, thus affecting performance and results. 

Therefore, a combination of sports training and psychological counseling resources should be used to observe the performance of fin swimmers in simulated high-stress competition situations to immediately identify and correct their psychological or physical deficiencies. This can improve athletes’ psychological deficiencies, strengthen their psychological quality, reduce the risk of mental health hazards, and improve the stability of athletic performance and results.

### 5.2. Effect of Imagery Training on Physical Anxiety in Fin Swimmers

Imagery training helped the athletes to relax psychologically, effectively reducing anxiety and improving negative physical or psychological symptoms of anxiety and stress. The results of this study are consistent with those in the literature [25,29,32,60,61,62]. The evidence is not consistent with the study Hypothesis 2.

Imagery training can simulate the atmosphere of the competition, the movements and performance of competitors, and replay past memories through videos. Imagery training provides different perspectives on time and space, as if the learner is experiencing it firsthand. Through the guidance of and explanations from coaches, participants’ deficiencies are identified, problems are improved, emotions are stabilized, and stress resistance is increased. Once the core of the problem is found, improvement measures can be identified and suggested to athletes, enhancing athletes’ confidence and strengthening their sports performance as well as naturally improving the psychological problems of athletes when they participate in a competition.

Imagery training is effective in relaxing the body, reducing anxiety and nervousness. Although it only provides a small amount of relief from physical tension during the competition in terms of reducing the negative physiological phenomena of stomach pain and rapid heartbeat, it can still be an influential factor in an athlete’s performance.

Therefore, this study concluded that in addition to enhancing the level of engagement in imagery training, the format and intensity of imagery training simulations should be elevated, so that athletes can adapt to high-stress competition situations. This will enhance their adaptability to high-stress environments, strengthen their psychological quality, and improve their negative physical and psychological symptoms during competitions.

### 5.3. Effects of Imagery Training on the Athletic Performance of Fin Swimmers

Although imagery training helped improve the athletes’ performance during the competition, errors still occurred, resulting in poor performance. This finding is inconsistent with those in the literature [42,59,60,61,62,63]. The evidence is not consistent with the study hypothesis 3.

Imagery training employs playback to capture images of other people’s or athletes’ own movements, which are then systematically planned and designed to allow the learner to develop tactics, modify movements, and adjust and set training and competition plans. Thus, imagery training can be compared to a dream that is repeatedly played over and over again in an attempt to be embedded in the athlete’s mind to pursue perfect movement, behavior, and performance. However, individual performance and behavior can be affected by internal physiological factors, such as congenital conditions and a lack of training, as well as by external interference such as spectators, judges, or athletes during competition or even accidents.

Therefore, even though imagery training can improve the performance of fin swimming athletes as well as help them exceed the normal level of athletic performance and achieve the expected competition goals, mistakes can still occur.

Therefore, we believe that to strengthen the basic skills of athletes, in addition to improving their skills and athletic ability through physical characteristics, this experience can lead to greater stability and higher technical learning and athletic ability in athletes.

### 5.4. Correlation of Imagery, Physical Anxiety, and Athletic Performance

The athletes who underwent imagery training showed improved imagery abilities and consequently improved athletic performance, but the actual effect was limited. This finding is inconsistent with that in the literature [26,37,38,41,42,59,60,61,62,63]. The evidence is not consistent with the study hypotheses 4 and 5.

Imagery training uses images or videos, coupled with coaching and guidance, to enhance athletes’ adaptability to the competition environment, improve their individual technical proficiency, and increase performance. However, because many internal and external uncertainties exist in the competition venue, strengthening imagery ability can enhance athletic performance, but the impact is limited. Therefore, imagery showed a significantly lower correlation with athletic performance in this study.

Furthermore, clear and explicit imagery training can ingrain athletes’ memory, strengthen their body memory [39], enhance the operational speed of motor skills [30], and ultimately lead to perfect athletic performance and favorable competition results.

Therefore, overall, imagery training improves sports performance. Strengthening strategic and technical imagery can result in above-average performance, which in turn will lead to the achievement of predetermined goals and satisfaction with competition and sports performance outcomes.

Therefore, this study concluded that in addition to strengthening the level of engagement of imagery training in sports patterns, the planning of strategic and technical imagery training should be refined. This will improve self-confidence and increase the stability of athletic performance, which will lead to higher athletic performance and achievement.

## 6. Conclusions

In this study, the level of engagement in imagery training influenced the physical and psychological performance of the athletes. Combining sports training and psychological counseling resources to observe the performance of fin swimmers in simulated high-stress competition situations and identifying and correcting the deficiencies of athletes’ psychological or physiological status in a timely manner will facilitate the athletes’ adaptation to high-stress competition situations, increase the stability of their sports performance, and improve their self-confidence, which will in turn facilitate a higher level of sports performance and achievement. The research recommendations are as follows:

### 6.1. For Training Institutions and Coaches

Training of athletes requires long-term investment and planning. Improving the psychological aspects of athletes and stabilizing their performance can achieve favorable performance and victory. Therefore, training institutions should create a relaxed and joyful learning atmosphere, and coaches should strengthen strategic and technical imagery training and provide psychological counseling or guidance channels to enhance stress resistance, maintain positive motivation, and improve crisis management skills among athletes, thereby improving sports performance.

### 6.2. For Athlete Training

Sports training is long term and challenging, but it is also a self-challenge and represents advancement. The professional training process leads to the development of social interaction and crisis management skills. Therefore, athletes who are under stress should actively seek help and increase other leisure activities, as well as receive appropriate sports massage, to relieve fatigue and improve their physical and mental health.

### 6.3. Future Research

This study was conducted using a mixed research method with Taiwanese fin swimming athletes, and questionnaires were used to obtain athletes’ perceptions. In addition, although imagery training can improve physical anxiety and enhance athletic performance and satisfaction, athletes may choose to withdraw from the competition when faced with frustration. Furthermore, this study only focused on fin swimming athletes in Taiwan and did not include participants in other competitive or recreational sports.

Therefore, to overcome these limitations, follow-up studies should use other methods or analyses to confirm the study findings or obtain more in-depth answers. Other sports should be examined, and the perspectives of recreational sports participants should also be investigated.

## Figures and Tables

**Table 1:** Background of experts and scholars.

	Gender	Certificate Level	Education	Teaching Qualifications	Highest Achievement
National team coach	Female	National	Master degree	30	World Games
Lecturer	Female	National	Master degree	30	Asian Indoor Games
Assistant professor	Male	National	Ph.D.	19	Asian Championship
Issue	As found in the data analysis, imagery training has a certain effect on the imagery ability of fin swimmers. What do you think are the key factors for the change? As found in the data analysis, imagery training has a certain effect on the physical anxiety of fin swimmers. What do you think are the key factors for the change? As found in the data analysis, imagery training has a certain impact on the performance of fin swimmers. What do you think are the key factors for the change?				

**Table 2:** Validity analysis of questionnaire.

Facet	Sub-Facet	Issue	Cronbach’s α
Imagery ability	Strategy	Imagine prematch tactics.	0.95
		Imagine alternative methods.	0.95
		Imagine future revision goals.	0.95
		Imagine the details of the game.	0.95
	Proficiency	The game did not go well, and I still worked hard.	0.95
		Imagine maintaining a positive attitude after setbacks.	0.95
		Remain confident when imagination is difficult.	0.95
		Imagining yourself is the most persevering.	0.95
	Skill	Correct the shortcomings of sports technology in time.	0.95
		Enhance sports proficiency through imagination.	0.95
		Revise basic movements through imagination.	0.95
		Imagine performing the action successfully.	0.95
	Emotion	Imagine the positive emotions during sports and overcome the difficulties of the game.	0.95
		When I imagine working on a good project, I feel expectation and excitement.	0.95
		Imagine feeling excited during sports performance.	0.95
		I can imagine the mood when I have a good performance.	0.95
	Goal	Imagine a medal you won after the game.	0.95
		Imagine winning the game.	0.95
		Imagine other athletes, coaches, and parents encouraging me because of my good performance.	0.95
Physical anxiety		Anxious	0.70
		Nervous	0.70
		Body feeling tense	0.70
		Body relaxation	0.70
		Stomach tension	0.70
		Accelerated heartbeat	0.70
Athletic performance		Satisfied with performance	0.71
		Have reached the predetermined goal	0.70
		Performance beyond previous levels	0.71
		Zero mistakes in sports performance	0.70

**Table 3:** Background analysis.

Background		*n*	%
Gender	Male	27	49%
	Female	28	51%
Exercise age	1 down	10	18%
	1–3	12	22%
	4–5	17	31%
	5 up	16	29%
Usage frequency	Infrequent use	9	16%
	Occasional use	18	33%
	Frequent use	16	29%
	Normal use	12	22%

**Table 4:** Perception analysis of the effect of imagery training on imagery ability.

		Before Training	SD	After Training	SD	Deviation	Up/Down	Rank
Strategy	Imagine prematch tactics.	3.62	0.904	4.23	0.732	0.61	↑	1
	Imagine alternative methods.	3.64	0.762	4.25	0.808	0.61	↑	1
	Imagine future revision goals.	3.98	0.665	4.02	0.593	0.04	↑	3
	Imagine the details of the game.	4.26	0.655	4.14	0.667	−0.12	↓	9
Proficiency	The game did not go well, and I still worked hard.	4.75	0.440	4.51	0.658	−0.24	↓	13
	Imagine maintaining a positive attitude after setbacks.	3.77	0.640	3.72	0.701	−0.05	↓	7
	Remain confident when imagination is difficult.	3.94	0.718	3.84	0.751	−0.10	↓	5
	Imagining yourself is the most persevering.	4.11	0.725	3.96	0.706	−0.15	↓	10
Skill	Correct the shortcomings of sports technology in time.	3.79	0.885	3.95	0.742	0.15	↑	2
	Enhance sports proficiency through imagination.	4.56	0.502	4.21	0.725	−0.35	↓	14
	Revise basic movements through imagination.	4.09	0.714	4.07	0.728	−0.02	↓	6
	Imagine performing the action successfully.	4.25	0.731	4.25	0.763	0.00	-	4
Emotion	Imagine the positive emotions during sports and overcome the difficulties of the game.	4.19	0.878	4.11	0.699	−0.08	↓	8
	When I imagine working on a good project, I feel expectation and excitement.	4.43	0.665	4.21	0.796	−0.22	↓	12
	Imagine feeling excited during sports performance.	4.11	0.751	4.12	0.709	0.00	-	4
	I can imagine the mood when I have a good performance.	4.13	0.785	4.18	0.685	0.04	↑	3
Goal	Imagine a medal you won after the game.	4.11	0.934	4.02	0.813	−0.10	↓	5
	Imagine winning the game.	4.30	0.799	4.14	0.811	−0.16	↓	11
	Imagine other athletes, coaches, and parents encouraging me because of my good performance.	4.17	0.612	4.07	0.678	−0.10	↓	5

**Table 5:** Perception analysis of the effect of imagery training on physical anxiety.

	Before Training	SD	After Training	SD	Deviation	Up/Down	Rank
Anxious	3.47	0.953	3.09	0.912	−0.38	↓	4
Nervous	3.66	0.837	3.12	0.946	−0.54	↓	5
Body feeling tense	3.53	0.973	3.21	0.840	−0.32	↓	3
Body relaxation	3.00	0.974	3.61	0.959	0.61	↑	1
Stomach tension	3.72	0.750	3.05	0.931	−0.66	↓	6
Accelerated heartbeat	3.70	0.749	3.39	0.774	−0.31	↓	2

**Table 6:** Perception analysis of effects of imagery training on athletic performance.

	Before Training	SD	After Training	SD	Deviation	Up/Down	Rank
Satisfied with performance	3.47	0.799	3.68	0.711	0.21	↑	3
Have reached the predetermined goal	3.58	0.819	3.89	0.673	0.31	↑	2
Performance beyond previous levels	3.49	0.869	4.05	0.718	0.56	↑	1
Zero mistakes in sports performance	2.89	1.013	3.05	0.875	0.17	↓	4

**Table 7:** Analysis of the correlation between imaginative ability and physical anxiety on sports performance.

	Overall Physical and Mental Anxiety	Overall Imagery Ability	Strategy	Proficiency	Skill	Emotion	Goal
Satisfied with performance	0.039	0.347 *	0.457 **	0.312	0.410*	0.159	0.205
Have reached the predetermined goal	0.086	0.321	0.456 **	0.297	0.389 *	0.171	0.105
Performance beyond previous levels	−0.008	0.308	0.443 **	0.284	0.298	0.178	0.167
Zero mistakes in sports performance	−0.111	0.391 *	0.514 **	0.368 *	0.383 *	0.214	0.266
Satisfied with performance	0.120	0.155	0.149	0.115	0.278	0.000	0.144

* *p* < 0.05 ** *p* < 0.001.

## Data Availability

No data available.
